# Therapeutic Efficacy of C-Kit-Targeted Radioimmunotherapy Using 90Y-Labeled Anti-C-Kit Antibodies in a Mouse Model of Small Cell Lung Cancer

**DOI:** 10.1371/journal.pone.0059248

**Published:** 2013-03-14

**Authors:** Chisato Yoshida, Atsushi B. Tsuji, Hitomi Sudo, Aya Sugyo, Tatsuya Kikuchi, Mitsuru Koizumi, Yasushi Arano, Tsuneo Saga

**Affiliations:** 1 Diagnostic Imaging Program, Molecular Imaging Center, National Institute of Radiological Sciences, Chiba, Japan; 2 Department of Molecular Imaging and Radiotherapy, Graduate School of Pharmaceutical Sciences, Chiba University, Chiba, Japan; 3 Molecular Probe Program, Molecular Imaging Center, National Institute of Radiological Sciences, Chiba, Japan; University of Navarra, Spain

## Abstract

Small cell lung cancer (SCLC) is an aggressive tumor and prognosis remains poor. Therefore, the development of more effective therapy is needed. We previously reported that high levels of an anti-c-kit antibody (12A8) accumulated in SCLC xenografts. In the present study, we evaluated the efficacy of two antibodies (12A8 and 67A2) for radioimmunotherapy (RIT) of an SCLC mouse model by labeling with the ^90^Y isotope.

**Methods:**

^111^In- or ^125^I-labeled antibodies were evaluated *in vitro* by cell binding, competitive inhibition and cellular internalization assays in c-kit-expressing SY cells and *in vivo* by biodistribution in SY-bearing mice. Therapeutic efficacy of ^90^Y-labeled antibodies was evaluated in SY-bearing mice upto day 28 and histological analysis was conducted at day 7.

**Results:**

[^111^In]12A8 and [^111^In]67A2 specifically bound to SY cells with high affinity (8.0 and 1.9 nM, respectively). 67A2 was internalized similar to 12A8. High levels of [^111^In]12A8 and [^111^In]67A2 accumulated in tumors, but not in major organs. [^111^In]67A2 uptake by the tumor was 1.7 times higher than for [^111^In]12A8. [^90^Y]12A8, but not [^90^Y]67A2, suppressed tumor growth in a dose-dependent manner. Tumors treated with 3.7 MBq of [^90^Y]12A8, and 1.85 and 3.7 MBq of [^90^Y]67A2 (absorbed doses were 21.0, 18.0 and 35.9 Gy, respectively) almost completely disappeared approximately 2 weeks after injection, and regrowth was not observed except for in one mouse treated with 1.85 MBq [^90^Y]67A2. The area of necrosis and fibrosis increased depending on the RIT effect. Apoptotic cell numbers increased with increased doses of [^90^Y]12A8, whereas no dose-dependent increase was observed following [^90^Y]67A2 treatment. Body weight was temporarily reduced but all mice tolerated the RIT experiments well.

**Conclusion:**

Treatment with [^90^Y]12A8 and [^90^Y]67A2 achieved a complete therapeutic response when SY tumors received an absorbed dose greater than 18 Gy and thus are promising RIT agents for metastatic SCLC cells at distant sites.

## Introduction

Lung cancer is the leading cause of cancer-related death worldwide [1]. Small cell lung cancer (SCLC) is a distinct clinicopathologic entity that accounts for up to 20% of all lung cancers and is distinguished from non-small cell lung cancer by its rapid tumor doubling time, high growth fraction and early development of widespread metastases [2]. Although SCLC is very sensitive to chemotherapy and radiation therapy, the overall prognosis remains poor because of high recurrence or metastatic rates, and the poor response of refractory SCLC [2], [3]. The median survival for patients with recurrent SCLC is approximately six months [3].

Recently, many molecular targeted drugs have been developed that contribute to the improved survival of patients with several cancers [4]. SCLC highly express several molecules such as c-kit, c-Met and vascular endothelial growth factor [4]. The c-kit proto-oncogene encodes a 145-kDa transmembrane tyrosine kinase receptor consisting of an extracellular portion with five immunoglobulin-like domains: the first three contain a ligand binding site and the fourth and fifth domains are associated with receptor dimerization, the transmembrane portion, and the intracellular portion having kinase enzymatic activity [5], [6], [7]. Stem cell factor is a ligand for c-kit and its binding leads to receptor activation and triggers the activation of downstream signal pathways involved in cell growth, differentiation and development. C-kit is implicated in the rapid cell growth observed in SCLC and thus is considered a candidate target molecule for diagnostics and therapeutics of SCLC [8]. Imatinib, an inhibitor of c-kit-tyrosine kinase activity, is highly effective against chemotherapy-resistant gastrointestinal stromal tumors (GIST) [9], [10]. Although several preclinical studies reported that suppression of c-kit signaling inhibits SCLC cell growth [4], [11], phase II imatinib trials in patients with relapsed c-kit-positive SCLC reported no objective responses or sustained disease stabilization [12], [13], [14]. This suggests that progression may not depend on c-kit owing to a lack of activating mutations unlike GIST, despite high c-kit expression in SCLC [15], [16]. Although blockade of the c-kit pathway may not have a therapeutic effect on SCLC, c-kit may be a promising target for the selective delivery of therapeutic agents such as toxins and radioisotopes to c-kit positive SCLC tumor cells by means of carriers such as antibodies.

We previously reported that high levels of ^111^In-labeled anti-c-kit antibody, 12A8, accumulated in c-kit-expressing SCLC xenografts, while its accumulation was low in normal organs [17], [18]. Therefore, 12A8 has the potential to be used for radioimmunotherapy (RIT) by substituting γ-emitting ^111^In with β- or α-emitting radionuclides with suitable nuclear properties. The concept of RIT has been realized in clinics for the treatment of non-Hodgkin B cell lymphoma, using anti-CD20 antibody labeled with ^90^Y or ^131^I [19]. ^90^Y is a pure β-emitter with high energy (maximum energy, 2.3 MeV), long particle range (maximum range in water, 11.3 mm), an appropriate half-life (64.1 h) for RIT with IgG and suitable for RIT with an internalizing antibody [19], [20]. In the present study, we evaluated and compared the *in vitro* and *in vivo* properties of two radiolabeled anti-c-kit monoclonal antibodies, 12A8 and 67A2, and their use in experimental RIT of SCLC using ^90^Y-labeled antibodies.

## Materials and Methods

### Cells

A human SCLC cell line SY (Immuno-Biological Laboratories (IBL), Takasaki, Japan) that has high c-kit expression was maintained in RPMI1640 (Sigma, St. Louis, MO, USA) containing 5% fetal bovine serum (Sigma, St. Louis, MO, USA) in a humidified incubator maintained at 37°C with 5% CO_2_.

### Antibodies

Two anti-c-kit mouse monoclonal antibodies (12A8 [17] and 67A2) were purchased from IBL. Each antibody binds to a different epitope on the extracellular portion of c-kit: 12A8 binds to the fifth domain nearest to the transmembrane domain and 67A2 binds to the second domain. The equilibrium dissociation constant (Kd) of 12A8 and 67A2 is 1.06 and 0.26 nM, respectively, as measured by a surface plasmon resonance assay. 12A8 has strong neutralization and moderate antitumor activities but does not induce antibody-dependent cell cytotoxicity (ADCC) or complement-dependent cytotoxicity (CDC). 67A2 does not possess any of the above-mentioned activities (information supplied from the manufacturer).

### Radiolabeling of antibodies

Antibodies (12A8 and 67A2) were conjugated with *p*-SCN-Bz-CHX-A''-DTPA (DTPA; Macrocyclics, Dallas, TX, USA) as previously described [17], [18], and then DTPA-conjugated antibodies were purified using a Sephadex G-50 (GE Healthcare, Little Chalfont, UK) column. These DTPA-conjugated antibodies (20 µg) were mixed with 1.2 MBq of [^111^In]Cl_3_ or 9.25 MBq of [^90^Y]Cl_3_ in 0.5 M acetate buffer (pH 6.0), and the mixture was incubated for 30 min at room temperature. Radiolabeled antibodies were separated from free ^111^In or ^90^Y using a Sephadex G-50 column. The conjugation ratio of DTPA to both antibodies was estimated to be 1.1, as determined by cellulose acetate electrophoresis, and the specific activities of [^111^In]12A8, [^111^In]67A2, [^90^Y]12A8 and [^90^Y]67A2 were approximately 50, 50, 300 and 450 kBq/μg, respectively. The labeling yield was approximately 80% for ^111^In labeling and 65% to 97% for ^90^Y labeling, and the radiochemical purity exceeded 96%. 67A2 was also labeled with ^125^I using chloramine-T for internalization assay as previously described [17]. The specific activity of [^125^I]67A2 was approximately 500 kBq/μg.

### *In vitro* assay

Cell binding, competitive inhibition and internalization assays were conducted as previously described [17]. Briefly, in a cell binding assay, serially-diluted SY cells in PBS were incubated with the ^111^In-labeled antibody on ice for 60 min. After washing, the radioactivity bound to the cells was measured. The immunoreactivity of the ^111^In-labeled antibodies was estimated according to the method of Lindmo *et al* [21]. In a competitive inhibition assay, the ^111^In-labeled antibody was incubated with SY cells in the presence of varying concentrations of the unlabeled antibody on ice for 60 min. After washing, radioactivity bound to the cells was counted. Data were analyzed and the Kd was estimated using GraphPad Prism software (Graphpad Software, La Jolla, CA, USA). In an internalization assay, SY cells were preincubated in culture medium with ^125^I- or ^111^In-labeled antibody on ice for 60 min. After washing, collected cells were further cultured at 37°C or on ice in fresh medium without radiolabeled antibodies. At various time points, the supernatant and the cells were separated by centrifugation. Trichloroacetic acid was added to the supernatant on ice and then separated by centrifugation to determine the non-protein-bound fraction (supernatant) and protein-bound fraction (pellet). The cells were washed with acidic buffer and then separated by centrifugation to determine both the membrane-bound (supernatant) and internalized fraction (pellet).

### Biodistribution of ^111^In-labeled antibody

BALB/c-nu/nu mice (5 weeks old, CLEA Japan, Tokyo, Japan) were inoculated subcutaneously with 2×10^6^ SY cells in the left thigh. Mice (19 to 23 g in body weight) bearing SY tumors were intravenously injected with 37 kBq of ^111^In-labeled antibody. The injected protein dose was adjusted to 20 µg per mouse. At 1, 2, 4, 7 and 10 days after injection of ^111^In-labeled antibody, five mice at each time point were euthanized, and blood was obtained from the heart. The tumor and major organs were removed and weighed, and radioactivity counts were measured using a gamma counter. The data were expressed as the percentage of injected dose per gram of tissue (% ID/g) decay-corrected and normalized to a 20-g body weight mouse.

The tumor absorbed dose for ^90^Y-labeled antibodies was estimated from the biodistribution data of ^111^In-labeled antibodies. ^90^Y emits a β-particle with a mean emitted energy of 0.9331 MeV, thus the mean energy emitted per transition was calculated as 1.495×10^−13^ Gy kg (Bq s)^−1^ based on 1 eV is equal to1.60218×10^−19^ J [22]. Tumor uptake at various time points was plotted against time, from which the area under the curve (AUC) was calculated. The dose absorbed by the tumor up to 10 days after injection was estimated using AUC and mean energy emitted per transition as follows: AUC×1.495×10^−13^×injected activity. The absorbed dose to the red marrow in a 70-kg reference man was estimated from our biodistribution data using OLINDA/EXM version 1.0 software (Vanderbilt University, Nashville, TN, USA) as previously described [23].

### Radioimmunotherapy

Two weeks after inoculation of SY cells, mice received an intravenous injection of 0.74, 1.85 or 3.7 MBq of ^90^Y-labeled antibody (n = 5 for each group). Tumor size was 4.4±3.2 mm^3^ at the time of administration. The protein dose was adjusted to 20 µg for each preparation by adding unlabeled antibody. As negative controls, mice were intravenously injected with the unlabeled antibody (20 µg protein/mouse) or PBS alone (defined as untreated), respectively. The body weight and tumor growth were determined twice a week. Tumor volume (mm^3^) was calculated as width×height×depth (mm)/2. Mice were euthanized when the xenografted tumor volume reached more than 200 mm^3^. This experimental protocol was approved by the Institutional Animal Care and Use Committee of the National Institute of Radiological Sciences, and all animal experiments were conducted in accordance with the institutional guidelines regarding animal care and handling.

### Histological analysis

Tumor samples were removed 1 week after injection and fixed in 10% (v/v) neutral buffered formalin and embedded in paraffin for sectioning (n = 5 for each group). Sections (3 μm thickness) were stained with hematoxylin and eosin (H&E). Apoptotic cells in the tumor were detected by terminal deoxynucleotidyl transferase-mediated deoxyuridine triphosphate nick-end labeling (TUNEL) staining using an ApopTag Plus Peroxidase *In Situ* Apoptosis Detection kit (Chemicon International, Temecula, CA) as previously described [24]. We quantified TUNEL-stained cells in at least five randomly selected fields at 400×magnification.

### Statistical analysis

Tumor growth data were analyzed by two-way repeated-measures ANOVA with the Student–Newman–Keuls method multiple comparison test. Apoptotic cell data were analyzed by Student's *t*-test. A value of *P*<0.05 was considered statistically significant.

## Results

### *In vitro* characterization of ^111^In-labeled antibodies

**Figure 1:**
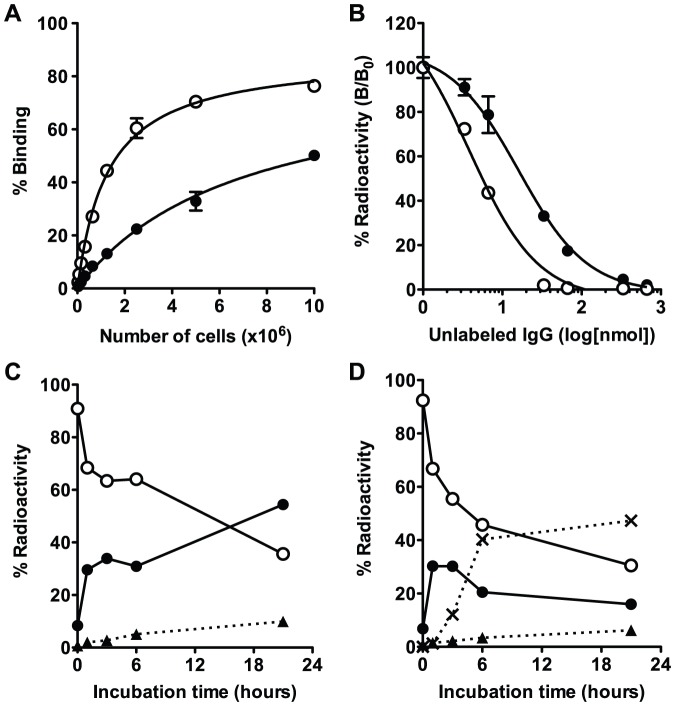
*In vitro* assay of [^111^In]12A8, [^111^In]67A2 and [^125^I]67A2. (**A**) Cell binding assay for [^111^In]12A8 (closed circles) and [^111^In]67A2 (open circles). (**B**) Competitive inhibition assay for [^111^In]12A8 (closed circles) and [^111^In]67A2 (open circles). Internalization assay for [^111^In]67A2 (**C**) and [^125^I]67A2 (**D**). Changes in % of total radioactivity for each fraction are plotted against incubation time at 37°C (closed circles, internalized fraction; open circles, membrane-bound fraction; closed triangles, protein-bound fraction in the culture medium; cross marks, non-protein-bound fraction in the culture medium).

From the cell binding assay, binding of [^111^In]12A8 and [^111^In]67A2 using 1×10^7^ SY cells was 50 and 76%, respectively (Fig. 1A). Binding of [^111^In]67A2 to SY cells was higher compared with that of [^111^In]12A8, especially at low cellular concentrations. The immunoreactive fraction of [^111^In]12A8 and [^111^In]67A2 was 83 and 88%, respectively. From the competitive inhibition assay, the Kd of [^111^In]12A8 and [^111^In]67A2 was estimated to be 8.0 and 1.9 nM, respectively (Fig. 1B). We examined the temporal change of radioactivity of [^111^In]67A2 and [^125^I]67A2 in the subcellular fraction (Fig. 1C and D). Radioactivity in the cell membrane-bound fraction of [^111^In]67A2 and [^125^I]67A2 rapidly decreased with time. In the case of [^111^In]67A2, the radioactivity of the internalized fraction increased with time, reaching approximately 55% after a 20-h incubation at 37°C (Fig. 1C). In contrast, internalized radioactivity of [^125^I]67A2 temporarily increased up to 3 h but thereafter decreased gradually. Radioactivity of the non-protein-bound fraction in the culture medium increased with time, reflecting dehalogenation of the ^125^I-labeled antibody in cells (Fig. 1D). When cells were incubated on ice, the membrane-bound fraction did not change and internalization was not observed for at least 3 h (data not shown). These results of the internalization assay were similar to those with 12A8 in our previous study [18].

### Biodistribution of [^111^In]12A8 and [^111^In]67A2

Long-term biodistribution experiments of [^111^In]12A8 and [^111^In]67A2 were conducted in nude mice bearing SY tumors from days 1 to 10 after injection (Tables 1 and 2). [^111^In]12A8 accumulated in tumor cells at 7.3±1.3% ID/g at day 1, and a peak value of 18.9±2.9% ID/g was obtained at day 4 (Table 1). The tumor uptake of [^111^In]67A2 was 15.7±0.9% ID/g at day 1, and a peak value of 31.5±7.7% ID/g was obtained at day 4 (Table 2). The AUC of [^111^In]67A2 was 1.7 times higher than that of [^111^In]12A8. The uptake of radiolabeled antibodies in major organs was low and decreased gradually with time, similar to that reported in previous studies for radiolabeled IgGs specific for other antigens [25], [26]. The tumor-to-blood ratio of [^111^In]12A8 and [^111^In]67A2 at day 1 was 0.6 and 0.9, respectively, and reached peak values of 3.6 and 4.0, respectively, by day 10.

**Table 1:** Biodistribution of [^111^In]12A8 in nude mice bearing SY xenograft.

	Day 1	Day 2	Day 4	Day 7	Day 10
Blood	12.5±0.7	11.5±2.6	7.6±0.7	3.7±1.2	3.7±0.9
Lung	3.0±0.1	3.3±0.6	2.5±0.4	1.6±0.3	1.8±0.6
Liver	5.3±0.5	4.8±0.8	4.0±0.5	4.0±0.6	2.7±0.5
Spleen	2.8±0.1	3.4±1.1	2.3±0.7	2.0±0.4	2.0±0.4
Stomach	0.6±0.1	0.5±0.2	0.5±0.1	0.3±0.1	0.2±0.1
Intestine	1.1±0.1	1.2±0.3	0.8±0.1	0.5±0.1	0.5±0.2
Kidney	8.7±0.5	8.3±1.4	5.3±0.4	3.6±0.6	2.6±0.6
Muscle	0.9±0.1	0.9±0.2	0.6±0.1	0.4±0.0	0.4±0.1
Bone	1.2±0.2	1.4±0.3	1.3±0.2	0.6±0.1	1.2±0.6
Tumor	7.3±1.3	16.7±1.5	18.9±2.9	11.1±2.1	13.4±5.7

Data are expressed as decay-corrected % ID/g±SD normalized to a 20-g body weight mouse.

**Table 2:** Biodistribution of [^111^In]67A2 in nude mice bearing SY xenograft.

	Day 1	Day 2	Day 4	Day 7	Day 10
Blood	17.0±2.8	13.7±0.5	10.2±0.5	4.5±1.6	4.4±2.0
Lung	4.2±0.5	4.2±0.7	3.4±0.7	1.6±0.5	1.7±0.6
Liver	4.7±0.3	4.9±0.7	4.1±0.7	4.1±1.0	2.9±0.4
Spleen	3.5±0.4	3.2±0.4	2.7±0.4	2.4±0.7	3.0±0.4
Stomach	0.7±0.3	0.8±0.2	0.5±0.2	0.6±0.3	0.4±0.1
Intestine	1.4±0.1	1.4±0.1	1.1±0.1	0.8±0.4	0.5±0.1
Kidney	4.9±0.7	4.1±0.2	3.7±0.2	2.2±0.3	2.1±0.5
Muscle	1.0±0.2	1.0±0.2	0.9±0.2	0.4±0.1	0.5±0.1
Bone	1.5±0.3	1.2±0.2	1.3±0.2	0.8±0.4	0.8±0.2
Tumor	15.7±0.9	23.8±1.6	31.5±7.7	18.2±2.2	16.7±3.0

Data are expressed as decay-corrected % ID/g±SD normalized to a 20-g body weight mouse.

The dose absorbed by tumors was estimated based on the AUC of each ^111^In-labeled antibody from the biodistribution data. The dose absorbed by tumors treated with 0.74, 1.85 and 3.7 MBq of [^90^Y]12A8 was estimated to be 4.2, 10.5 and 21.0 Gy, respectively, and that of [^90^Y]67A2 was 7.2, 18.0 and 35.9 Gy, respectively. The absorbed dose to red marrow was estimated from our biodistribution data to be 0.5 mGy/MBq in a 70-kg reference man for both ^90^Y-labeled antibodies.

### Radioimmunotherapy

**Figure 2:**
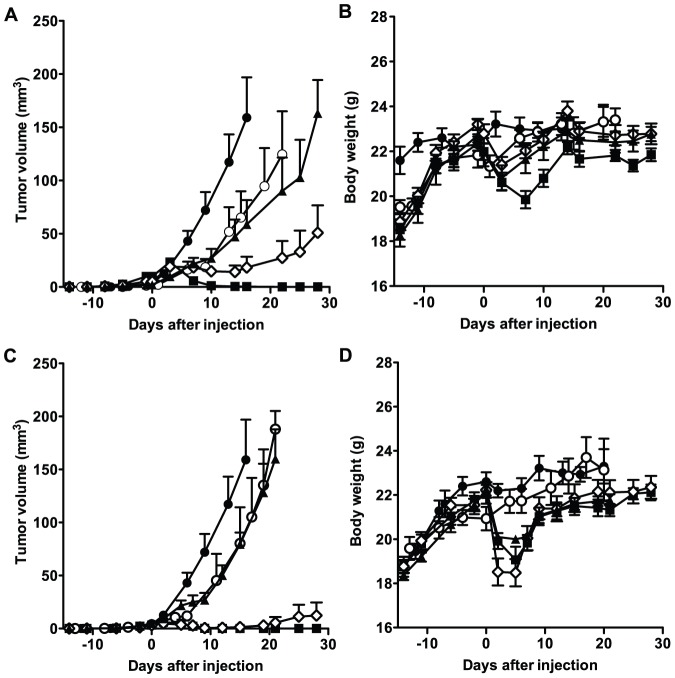
Growth curves of tumors and body weight of mice undergoing radioimmunotherapy with ^90^Y-lableled antibodies. Tumor growth curve (**A**) and body weight (**B**) for mice treated with [^90^Y]12A8. Tumor growth curve (**C**) and body weight (**D**) for mice treated with [^90^Y]67A2 (closed circles, untreated (PBS); open circles, unlabeled IgG alone; closed triangles, 0.74 MBq; open diamonds, 1.85 MBq; black squares, 3.7 MBq).

Following [^90^Y]12A8 treatment, the tumor volume in all groups increased until day 3 after injection, but thereafter decreased in the 3.7-MBq treatment group and completely disappeared around 2 weeks after injection (Fig. 2A). The administration of 1.85 MBq of [^90^Y]12A8 also suppressed tumor growth until around 2 weeks after injection, but thereafter tumor volumes started to increase again (Fig. 2A). In groups treated with unlabeled IgG alone and 0.74 MBq of [^90^Y]12A8, we observed tumor growth delay compared with the untreated (PBS) group (*P*<0.05) (Fig. 2A). [^90^Y]67A2 treatment showed a higher therapeutic effect compared with [^90^Y]12A8 at mid- to high-dose levels. The administration of 1.85 and 3.7 MBq of [^90^Y]67A2 markedly suppressed tumor growth, and tumors completely disappeared around 2 weeks after injection except for a single mouse treated with 1.85 MBq of [^90^Y]67A2. Although we observed tumor growth delay in the treatment group with 0.74 MBq of [^90^Y]67A2 compared with the untreated (PBS) group (*P*<0.05), there was no significant difference in tumor growth compared with the unlabeled IgG treatment group (Fig. 2C). The body weight of mice treated with [^90^Y]12A8 and [^90^Y]67A2 temporarily decreased by approximately 15%, but started to increase within the first week after treatment (Fig. 2B and D), and all mice tolerated the RIT experiment up to day 28 (the last day observed).

### Histological analysis

**Figure 3:**
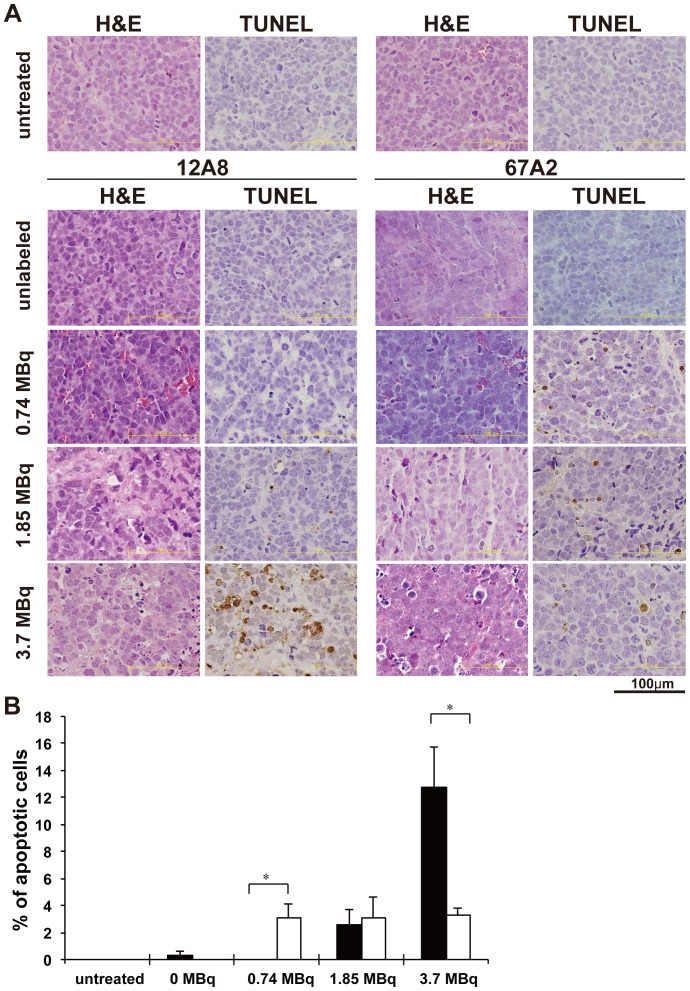
Histological analysis of tumors. (**A**) H&E and TUNEL stained tumor sections one week after injection of [^90^Y]12A8 and [^90^Y]67A2. (**B**) Quantification of apoptotic cells in tumors treated with [^90^Y]12A8 (black bars) and [^90^Y]67A2 (white bars). *P<0.01 (Student's *t*-test).

High numbers of mitotic cells were observed in H&E-stained sections of SY tumors from the untreated group (PBS), reflecting a high proliferation activity (Fig. 3A). Tumor sections obtained at day 7 after injection of [^90^Y]12A8 revealed areas of necrosis and fibrosis that increased in a dose-dependent manner (Fig. 3A). Necrosis and fibrosis were also observed in tumors treated with 1.85 and 3.7 MBq of [^90^Y]67A2, but not with unlabeled IgG alone and 0.74 MBq of [^90^Y]67A2 (Fig. 3A). On TUNEL-stained sections, apoptotic cells were rarely observed under untreated conditions (Fig. 3A and B). In tumors treated with [^90^Y]12A8, the percentage of apoptotic cells tended to increase with an increase in the dose of radioactivity (Fig. 3A and B). In contrast, in tumors treated with [^90^Y]67A2, the same level of apoptotic cells was observed for 0.74 to 3.7 MBq without a dose-dependent increase (Fig. 3A and B).

## Discussion

SCLC is an aggressive tumor and the 2-year survival rate for patients with limited-stage and extensive-stage diseases is between 20 to 40% and 2 to 5%, respectively [27], [28], [29]. Therefore, additional effective anticancer therapy is required, especially for patients with extensive-stage disease. SCLC expresses high levels of c-kit that enables rapid cell growth and thus is a key candidate molecule for diagnosis and therapeutics in SCLC [30], [31], [32]. We previously demonstrated that high levels of anti-c-kit antibody [^111^In]12A8 accumulated in c-kit-expressing tumors, but not in normal organs [17], [18]. Since SCLC is sensitive to radiation, RIT using 12A8 labeled with cytotoxic radionuclides such as ^90^Y or ^131^I has the potential to be a more effective treatment for SCLC than the current treatments. In the present study, we employed an additional antibody 67A2 that has a higher affinity for c-kit compared with 12A8, and evaluated the therapeutic efficacy of ^90^Y-labeled 12A8 and 67A2 in an SCLC mouse model.

*In vitro* characterization of ^111^In-labeled 12A8 and 67A2 demonstrated that [^111^In]67A2 had four times higher binding affinity than [^111^In]12A8, as expected. Although the epitope recognized by 67A2 is different from that of 12A8, the internalization assay showed that 67A2 was rapidly internalized after binding to c-kit similar to that seen for 12A8 [17]. As reported for 12A8, following c-kit binding and internalization, radiolabeled 67A2 was metabolized and ^125^I activity was cleared from the cells, while ^111^In activity remained, indicating metal radionuclides are suitable for imaging (^111^In) and therapy (^90^Y) using 67A2 to target tumor cells.

To determine if the antibodies could be used for RIT of c-kit-expressing tumors, we evaluated the long-term biodistribution of ^111^In-labeled antibodies, from which the tumor absorbed dose was estimated for ^90^Y-labeled corresponding antibodies. [^111^In]67A2, having four times higher binding affinity than [^111^In]12A8, showed a higher tumor uptake at every time point studied. AUC of tumor time activity curve was 1.7 times greater for [^111^In]67A2 than for [^111^In]12A8, demonstrating a higher tumor absorbed dose using [^90^Y]67A2.

Next, we labeled both antibodies with ^90^Y and conducted experimental RIT in mice bearing SY tumors. Administration of 3.7 MBq of [^90^Y]12A8 or 1.85 and 3.7 MBq of [^90^Y]67A2 almost completely suppressed tumor growth. Comparison of absorbed dose calculation indicated that tumors receiving an absorbed dose greater than 18 Gy achieved a complete response in SY tumors. RIT was tolerated in mice, which only developed transient body weight loss. These findings suggest that the ^90^Y-labeled antibodies have the potential to deliver a lethal dose of radiotherapy to c-kit-expressing SCLC. The dose-limiting organ for RIT with IgG is commonly the red marrow, and consideration of the absorbed dose to the red marrow is important in determining the therapeutic radiation dose in patients [33]. In the present study, the absorbed dose estimated from our biodistribution data was 0.5 mGy/MBq in a 70-kg reference man for both ^90^Y-labeled antibodies;≤2 Gy is commonly considered to be a safe radiation dose [33], indicating that the estimated maximum therapeutic dose is 4 GBq. However, Tolvanen *et al*. reported that there were discrepancies between the absorbed doses estimated from rat- and human-derived data [23]. In addition, the anti-c-kit antibodies 12A8 and 67A2 bind to human c-kit but not to murine c-kit. Therefore, we need a future clinical study with ^111^In-labeled 12A8 and 67A2 in patients to estimate accurate dosimetry. 38% to 78% of SCLC specimens and 42% to 52% of SCLC cell lines have high expression of c-kit [30], [34]; therefore, more than approximately 40% of SCLC patients could potentially benefit from c-kit targeted radioimmunotherapy. This should be further validated in future clinical studies.

Interestingly, [^90^Y]12A8 suppressed tumor growth in a dose-dependent manner, while [^90^Y]67A2 behaved as if it required a threshold dose to be effective. No significant difference in tumor growth was found between unlabeled IgG alone and 0.74 MBq [^90^Y]67A2 treatment groups although 0.74 MBq [^90^Y]67A2 gave a tumor absorbed dose of 7.2 Gy, which was 1.7 times higher than that in 0.74 MBq [^90^Y]12A8. Histological analysis showed that the area of necrosis and fibrosis reflected the RIT effect. In contrast, the induction pattern of apoptosis was different between the two ^90^Y-labeled antibodies. Although it is not clear whether the difference in apoptosis induction is related to the different pattern of RIT effect, the differences in the biological characteristics of the antibodies may have caused this discrepancy. According to the manufacturer's information, 12A8 has a strong neutralization activity and mild anti-tumor activity, while 67A2 has neither, and both antibodies do not possess ADCC and CDC activities. Although there is no direct evidence that c-kit is involved in the apoptosis pathway, c-kit knockdown using short hairpin RNA induced apoptosis in breast cancer cells [35] and c-kit depletion by neutralizing antibody resulted in greatly increased apoptosis in differentiating spermatogonia in mice [36]. These findings raise the possibility that combined c-kit depletion with radiation therapy could induce synergistic effects, resulting in suppressing tumor growth using a low dose of [^90^Y]12A8 despite its lower absorbed dose compared with [^90^Y]67A2. As patients generally receive high doses in radical RIT, our findings suggest that high affinity is a very important characteristic for antibody used in RIT in a clinical setting, although we cannot exclude the possibility that other properties could affect RIT efficacy. It would be interesting to assess the possibility that therapeutic effects could be augmented by other biological character(s) of the antibody in future studies.

Although RIT has the potential to enhance the effect of antibody therapy in many types of tumors, in clinical use radioimmunotherapy to date has been effective only for hematological malignancies, but not for non-hematological malignancies [19], [37], [38]. Since it is difficult for antibodies to penetrate into entire solid tumors, radiolabeled antibodies cannot deliver a lethal dose to all the tumor cells within a solid tumor. However, since radiolabeled antibodies can systemically deliver cytotoxic radionuclides to tumor sites, RIT for solid tumors is generally considered suitable to treat small-sized metastatic tumors (less than several cm in diameter) but not bulky primary tumors (more than several cm in diameter). In this regard, SCLC, which has a high risk of widespread metastases but which is relatively radiosensitive, may be a good candidate for RIT. Although patients with extensive-stage SCLC traditionally have not received radiotherapy, Slotman *et al*. reported that prophylactic cranial irradiation in patients with extensive-stage SCLC resulted in a significant reduction of brain metastasis incidence and improvement of survival [39]. Therefore, [^90^Y]12A8 and [^90^Y]67A2 could be effective for the treatment of metastatic cancer cells in distant sites and may have the potential to reduce the incidence of metastases besides brain in SCLC, especially during the extensive stage of disease.

## Conclusion

We evaluated two radiolabeled anti-c-kit antibodies 12A8 and 67A2 for their possible application for RIT of SCLC. The affinity of [^111^In]67A2 was four times higher than that of [^111^In]12A8, and tumor uptake of [^111^In]67A2 was 1.7 times higher than that of [^111^In]12A8. At middle to high doses of ^90^Y-labeled antibodies, tumor control simply reflected the tumor absorbed dose, where a tumor absorbed dose greater than 18 Gy could induce almost complete regression of the SCLC xenograft. This suggests that SCLC may be a good target of RIT with ^90^Y-labeled anti-c-kit antibodies. [^90^Y]12A8 and [^90^Y]67A2 are promising RIT agents for extensive-stage SCLC.
